# The Contribution of Functional Near-Infrared Spectroscopy (fNIRS) to the Study of Neurodegenerative Disorders: A Narrative Review

**DOI:** 10.3390/diagnostics14060663

**Published:** 2024-03-21

**Authors:** Ioannis Liampas, Freideriki Danga, Panagiota Kyriakoulopoulou, Vasileios Siokas, Polyxeni Stamati, Lambros Messinis, Efthimios Dardiotis, Grigorios Nasios

**Affiliations:** 1 Department of Neurology, University Hospital of Larissa, Faculty of Medicine, School of Health Sciences, University of Thessaly, 41100 Larissa, Greece; vsiokas@med.uth.gr (V.S.); tzeni_0@yahoo.gr (P.S.); edar@med.uth.gr (E.D.); 2 Department of Speech and Language Therapy, School of Health Sciences, University of Ioannina, 45500 Ioannina, Greece; errikadg@gmail.com (F.D.); grigoriosnasios@gmail.com (G.N.); 3 School of Medicine, University of Patras, 26504 Rio Patras, Greece; panagiotakyriak@yahoo.com; 4 Laboratory of Neuropsychology and Behavioral Neuroscience, School of Psychology, Aristotle University of Thessaloniki, 54124 Thessaloniki, Greece; lmessinis@psy.auth.gr

**Keywords:** Alzheimer’s disease, mild cognitive impairment, frontotemporal lobar degeneration, Parkinson’s disease, amyotrophic lateral sclerosis

## Abstract

Functional near-infrared spectroscopy (fNIRS) is an innovative neuroimaging method that offers several advantages over other commonly used modalities. This narrative review investigated the potential contribution of this method to the study of neurodegenerative disorders. Thirty-four studies involving patients with Alzheimer’s disease (AD), mild cognitive impairment (MCI), frontotemporal dementia (FTD), Parkinson’s disease (PD), or amyotrophic lateral sclerosis (ALS) and healthy controls were reviewed. Overall, it was revealed that the prefrontal cortex of individuals with MCI may engage compensatory mechanisms to support declining brain functions. A rightward shift was suggested to compensate for the loss of the left prefrontal capacity in the course of cognitive decline. In parallel, some studies reported the failure of compensatory mechanisms in MCI and early AD; this lack of appropriate hemodynamic responses may serve as an early biomarker of neurodegeneration. One article assessing FTD demonstrated a heterogeneous cortical activation pattern compared to AD, indicating that fNIRS may contribute to the challenging distinction of these conditions. Regarding PD, there was evidence that cognitive resources (especially executive function) were recruited to compensate for locomotor impairments. As for ALS, fNIRS data support the involvement of extra-motor networks in ALS, even in the absence of measurable cognitive impairment.

## 1. Introduction

The number of older adults diagnosed with neurodegenerative disorders is rapidly growing. Neurodegenerative disorders are characterized by progressive neuronal loss and constitute the aftereffect of labyrinthine genetic and environmental interactions [1]. Their categorization is based on cardinal clinical manifestations (e.g., neurocognitive or movement disorders), spatial patterns of brain involvement (e.g., frontotemporal, extrapyramidal, temporoparietal neurodegeneration), or molecular pathology (e.g., α-synucleinopathies, β-amyloidoses, Tau-opathies, disorders associated with pathological formations of the TDP-43 protein) [2]. The lack of adequate and effective management combined with the economic and psychological burden of neurodegenerative disorders on patients and caregivers highlights the need for early diagnosis and effective preventive strategies [3,4]. 

However, the remarkable clinical heterogeneity along with the considerable clinico-pathological overlap of neurodegenerative disorders often makes their differential diagnosis quite challenging [5]. To establish an accurate diagnosis, modern neuroimaging techniques providing both structural (pathological and anatomical information) and functional (data on brain activation) information are often capitalized on. The combination of these techniques allows a comprehensive examination of brain pathology and has—to a certain extent—replaced the need for post-mortem brain studies (autopsies) [6,7]. Not only do these techniques serve as an adjunct to the diagnostic process, but they also contribute to the detection of the neuro-anatomical correlations of motor, cognitive, and behavioral changes, progressively becoming an integral part of clinical evaluation and research [8].

It is known that compensatory mechanisms in individuals with neurodegenerative disorders either recruit intact neural circuits of adjacent brain regions or activate existing neural networks to preserve cognitive and motor functioning [9]. These compensatory mechanisms co-occur with the onset of neuronal loss in the early stages of neurodegeneration [10]. The existence of a crucial breakpoint is hypothesized during the course of neurodegeneration, whereby the early pattern of neural compensation (maintenance of clinical performance) is succeeded by the more typical pattern of neurodegeneration (clinical impairment). This sequence is often captured by functional neuroimaging as increased cerebral perfusion (attributed to the build-up of neurovascular compensatory mechanisms accounting for higher metabolic needs that allow the preservation of normal functions) followed by decreased brain perfusion (subsequent failure of compensatory responses) with progressively greater diminution [11]. Among the available modalities, functional near-infrared spectroscopy (fNIRS) may serve as a non-invasive, low-cost, portable, easy-to-use diagnostic tool in the identification of early neurodegenerative alterations and subclinical compensatory responses. Brain activity is quantified in fNIRS by capturing hemodynamic responses. Evidence for both hypo- and hyper-activation (hypo- and hyper-perfusion) has been reported in prodromal disease stages; the latter is suggestive of compensatory responses in which alternate brain networks are recruited to counteract neurodegeneration [12,13].

### Functional Near-Infrared Spectroscopy

Functional near-infrared spectroscopy (fNIRS) is an optical, non-invasive neuroimaging technique developed by Jöbsis in 1977 to study the behavior of cytochrome c oxidase in vivo [14]. Later, it was found that the application of infrared light in the near range of 700–1300 nm offers good visibility of tissue oxygenation, which laid the foundations for the utilization of this method in the study of animal and human brains [15].

fNIRS depends on “neurovascular coupling”; neuronal activation during a task is associated with vasodilation and increased blood flow [16], followed by an increase in the concentration of oxyhemoglobin and a simultaneous decrease in the concentration of deoxyhemoglobin ensue [16]. fNIRS uses light rays close to the visible range (or optical window), which are emitted from a light source (source/light-emitting diode) to the skull and are subsequently captured by a photodetector that collects the scattered rays and measures the degree of light attenuation and absorption [17]. During this process, chromophores of the neural tissue absorb light more strongly than surrounding tissues [17]. The absorption of infrared light as a function of wavelength is different for oxyhemoglobin and deoxyhemoglobin molecules; the detection of changes in the relative concentrations of light-absorbing chromophores allows fNIRS to capture energy metabolism in the brain [16]. 

The utilization of fNIRS in clinical practice has rapidly increased over the last few decades for the functional study of the human brain. Compared to other functional neuroimaging modalities, such as positron emission tomography (PET), functional magnetic resonance imaging (fMRI), single-photon emission computed tomography (SPECT), magnetoencephalography (MEG), and electroencephalography (EEG), fNIRS exhibits several important advantages: the portability of the device (which allows the measurement of brain activity in various settings), its low cost, its high tolerance by patients, its compatibility with other therapeutic devices (e.g., electroencephalogram—EEG), the high temporal resolution of the data obtained (with a maximum sampling rate approaching 100 Hz), and the low interference of head movements with cerebral signals [8,18,19]. fNIRS is, therefore, a potentially useful alternative functional neuroimaging technique for diagnostic and rehabilitation purposes related to various acquired or inherited neurological conditions (such as neurodevelopmental syndromes, epilepsy, neurodegenerative diseases, traumatic brain injuries, and strokes), as well as psychiatric disorders (such as mood disorders, developmental disorders, and schizophrenia [20,21]). 

## 2. Methods

We searched for clinical studies that used fNIRS either alone or in combination with other imaging modalities to obtain task-related and resting-state cortical activation data in patients diagnosed with the following common neurodegenerative entities: MCI, AD, FTD, PD, and ALS. We focused on studies involving both a group of participants with a neurodegenerative disorder and a comparator group of healthy controls (HC). Uncontrolled studies, controlled studies assessing other neurological conditions, non-observational studies (including reviews, meta-analyses, case reports, editorials, commentaries, viewpoints, and so on), study protocols, book chapters, reviews, and studies not published in English were excluded. Two authors (F.D. and I.L.) independently performed the literature search, data extraction, and interpretation. Potential dissensions were resolved by a third author (G.N.). The literature search was conducted in PubMed and Google Scholar. The search terms included “fNIRS’’ AND [‘‘neurodegenerative diseases” OR “Alzheimer’s disease” OR “Parkinson’s disease” OR “mild cognitive impairment” OR “amyotrophic lateral sclerosis” OR “frontotemporal degeneration”]. 

## 3. Results

### 3.1. fNIRS in MCI

MCI lies on the normal cognition–dementia continuum of cognitive decline [22,23]. The construct of MCI has been specifically designated to describe an early stage of clinically measurable cognitive impairment that, however, does not interfere with the daily activities of an individual [24]. Apart from cognitive impairment, greater neuropsychiatric burden and accelerated courses of cognitive decline and conversion into dementia have been related to this minor neurocognitive entity [25,26,27,28]. The concept of MCI has both research and clinical applications, allowing physicians to recruit individuals at high risk of progressing to dementia and apply early preventive strategies [3]. 

A total of 16 articles comparing individuals with MCI and HC were retrieved (Table 1). Ung and colleagues showed greater bilateral prefrontal activation in individuals with MCI during a visuospatial working memory task [29]. Moreover, differences were increasingly steeper with increasing task difficulty, leading to the speculation that MCI patients could handle low working memory loads without the need to compensate, but compensatory mechanisms were recruited at higher levels of difficulty. Similarly, Kim and colleagues reported higher activation of the prefrontal cortex in individuals with MCI during a verbal fluency task, suggesting that MCI patients used compensatory mechanisms and required more energy than the HC to perform the same task [30]. Yoon and colleagues found greater activation of the right prefrontal cortex in patients with non-amnestic MCI and hypoactivation in those with amnestic MCI during the Stroop test [31]. The authors hypothesized that there were active compensatory mechanisms only in the former group. Yang and colleagues reported hypoactivation of the left but not the right prefrontal cortex in MCI individuals during verbal fluency, Stroop, and N-Back tasks [32,33]. The authors theorized that only the right prefrontal cortex in those with MCI can recruit existing neural compensatory mechanisms. Finally, Yap and colleagues observed higher (though non-significant) activation of the right prefrontal cortex in individuals with MCI during a verbal fluency task and reached similar conclusions to those reported by Yang and colleagues [34]. Overall, these studies support the concept that the prefrontal cortex of individuals with MCI may engage neural compensatory mechanisms to support declining brain functions. The right prefrontal cortex appears to be of crucial importance in this process; the rightward shift of prefrontal recruitment has been suggested to compensate for the loss of the left prefrontal capacity in the course of healthy and pathological aging [35]. Disparities between different MCI subtypes are to be expected; however, additional research is required to better understand these differences.

On the other hand, results indicative of the failure of compensatory mechanisms in MCI have been published as well. Yeung and colleagues showed that contrary to the HC, individuals with MCI did not exhibit significant bilateral frontal activation with high working memory load during the 0-, 2-Back task [36]. Consequently, the authors suggested a failure of the MCI group to deploy compensatory efforts in response to increasing task demands. Niu and colleagues found decreased activation of the left dorsolateral prefrontal, right supplementary motor, and left superior temporal regions in individuals with MCI during the 0-, 1-Back task [37]. The authors deduced that the MCI group failed to recruit sufficient frontotemporal resources for task performance and to show the expected task-related activation exhibited by the HC. Similarly, Haberstumpf and colleagues reported that MCI participants exhibited reduced bilateral parietal activation during the clock-hand angle discrimination task, suggesting a failure to recruit compensatory neural mechanisms [38]. Moreover, Li and colleagues assessed participants with MCI and HC on the digit verbal span task and reported reduced activation of frontal and bilateral parietal cortices among those with MCI [39]. Katzorke and colleagues examined individuals with MCI and HC on the verbal fluency task and found decreased activation of bilateral inferior frontotemporal regions in MCI [40]. Uemura and colleagues assessed older adults with amnestic MCI and HC on memory encoding and delayed retrieval and observed reduced bilateral dorsolateral prefrontal activation in the MCI group during the memory retrieval task [41]. Finally, Arai and colleagues evaluated participants with amnestic MCI and HC on the verbal fluency task and documented lower activation of the right parietal area in those with MCI [42]. 

Based on the above, many authors have argued that the lack of hemodynamic responses in the respective cortical areas during specific neuropsychological tasks may serve as early biomarkers of neurodegeneration. Heterogeneity among published studies should most likely be attributed to the involvement of participants with diverse MCI subtypes, different levels of cognitive impairment, and disparate underlying neuropathological alterations. Additional heterogeneity is probably introduced by cognitive assessments, considering that different neuropsychological tasks target different cognitive domains and bring in different cognitive workloads. Consequently, it is almost impossible to compare—let alone synthesize—the results of published articles involving participants with MCI and HC. Future studies are required to create more homogeneous groups of MCI individuals in order to reveal distinct patterns of cortical hyper- or hypoactivation that may reflect each underlying pathology/MCI subtype at different stages of cognitive decline (existing compensatory mechanisms vs. failure of compensatory responses on the grounds of more advanced neurodegeneration. 

Of note, among the articles retrieved, three focused on functional brain connectivity. Nguyen and colleagues evaluated MCI patients and HC during a resting state and on the oddball, 1-Back, and verbal fluency tasks [43]. Individuals with MCI had higher right and inter-hemispheric connectivity than that of the HC during the resting state and lower left and inter-hemispheric connectivity during the verbal fluency task. Moreover, significantly greater inter-hemispheric than intra-hemispheric connectivity was reported in the HC group during the verbal fluency task—no difference between the inter- and intra-hemispheric connectivity was found in the MCI group. Niu and colleagues assessed participants with amnestic MCI and HC during a resting state and reported disrupted dynamic brain connectivity with increased variability in those with MCI [44]. Finally, Wang and colleagues examined individuals with MCI and HC during walking tasks [45]. Although no differences were detected during the walking-only tasks, connection strength was greater in the HC than in the MCI patients during more difficult dual task (more complex cognitive activities elicited greater differences). Moreover, connection strength changes with escalating difficulty distinguished those with MCI from the HC. Based on the above, functional connectivity evaluated via fNIRS during a resting state and in cognitive and dual (+walking) tasks could contribute to the screening for cognitive impairment.

**Table 1:** fNIRS studies involving older adults with MCI and HC.

Study	Participants	Clinical Scores	Mean Age in Years ± SD	Tasks [Corresponding Functions]	fNIRS Data [Mentioned Involved Areas]	Conclusions
Wang et al., 2022 [45]	MCI (*n* = 16) HC (*n* = 38)	MMSE: 22.9 ± 2.1 (MCI), 28.4 ± 1.6 (HC) MoCA: 17.6 ± 2.0 (MCI), 25.7 ± 2.3 (HC)	69.7 ± 6.5 (MCI) 67.9 ± 7.4 (HC)	Walking-only task [Automaticity and basic motor functions (gait speed, stride time, and stride time variability)] Dual-Task Walking and counting—easy condition [Walking: Automaticity and basic motor functions (gait speed, stride time, and stride time variability). Counting forward: Working memory and short-term memory)] Dual-Task Walking and subtracting—difficult condition [Walking: Automaticity and basic motor functions (gait speed, stride time, and stride time variability). Subtracting: Working memory, executive functions]	No significant differences in overall functional connectivity between the two groups during walking-only task. The connection strength of regions of interest was greater in HC than MCI during the dual-task walking—difficult condition. Connection strength changes with escalating difficulty distinguished those with MCI from HC.	The normal population has the ability to overcome the interference of more difficult cognitive sub-tasks in gait—the ability of people with cognitive impairment is relatively insufficient.
Haberstumpf et al., 2022 [38]	MCI (*n* = 59) HC (*n* = 59)	MMSE: 28.9 ± 1.2 (MCI), 29.3 ± 0.9 (HC) DemTect: 15.2 ± 2.3 (MCI), 16.1 ± 2.1 (HC)	74.1 ± 1.6 (MCI) 73.6 ± 1.5 (HC)	Clock-hand-angle discrimination task [Visuospatial processing skills, attention, and working memory]	Parietal cortex: Bilaterally reduced activation in MCI. Increased activity in the right compared to the left hemisphere in both groups.	Hemodynamic deficits indicate that MCI patients exhibit no compensation within the parietal cortex.
Ung et al., 2020 [29]	MCI (*n* = 12) Mild AD (mAD) (*n* = 18) HC matched for age, gender and educational level. (*n* = 31)	MMSE: 26.0 ± 3.1 (MCI), 21.2 ± 3.6 (mAD), 28.7 ± 1.5 (HC) CDR: 0.5 (MCI), 1.0 (mAD), 0.0 (HC)	73.1 ± 8.2 (MCI) 74.7 ± 10.0 (mAD) 72.6 ± 8.5 (HC)	Visuospatial working memory task based on «Neuro Recall» [Visuospatial Working Memory]	Bilateral prefrontal cortex: Increasing activation with increasing task difficulty in HC and MCI—even steeper increase in MCI. Little sign of increasing activation as task difficulty increases in AD.	MCI patients could handle a low visuospatial working memory load without the need to compensate. At higher levels of difficulty, compensatory mechanisms were recruited. AD patients could not recruit any compensatory mechanisms.
Yang et al., 2019 [32]	MCI (*n* = 15) HC matched for age and educational background (*n* = 9)	K-MMSE: 25.1 ± 2.3 (MCI), 27.2 ± 2.0 (HC)	69.3 ± 7.1 (MCI) 68.3 ± 4.7 (HC)	Stroop test [Executive functions (Inhibitory control and Cognitive flexibility)] N-Back [Working Memory] Verbal Fluency Task [Verbal ability, Executive functions (Updating of Working Memory, Inhibitory control, and Cognitive flexibility)]	This study investigated fNIRS biomarkers for distinguishing healthy control (HC) and MCI patients. Fifteen digital biomarkers from three brain regions (left, middle, right prefrontal cortex) were evaluated along with two image biomarkers (t-map, correlation map) during three mental tasks (N-back, Stroop, and verbal fluency task). This study was based on the same dataset as that in the study of Yang and Hong, 2019 (see below).	Convolutional neural network results trained via t-maps revealed the best accuracy, i.e., 90.62%, with the N-back task, and 85.58% trained with correlation maps. Investigation of sub-regions (i.e., left, middle, right prefrontal cortex) was better than examining the whole prefrontal cortex. The t-map and/or correlation map was recommended as an image biomarker for detecting MCI.
Yang and Hong, 2019 [33]	MCI (*n* = 15) HC matched for age and background education (*n* = 9)	K-MMSE: 25.1 ± 2.3 (MCI), 27.2 ± 2.0 (HC)	69.3 ± 7.1 (MCI) 68.3 ± 4.7 (HC)	Stroop test [Executive functions (Inhibitory control and Cognitive flexibility)] N-Back [Working Memory] Verbal Fluency Task [Verbal ability, Executive functions (Updating of Working Memory, Inhibitory control and Cognitive flexibility)]	This study investigated fNIRS biomarkers for distinguishing healthy control (HC) and MCI patients. Ten digital biomarkers from three brain regions (left, middle, right prefrontal cortex) were evaluated during three mental tasks (N-back, Stroop, and verbal fluency task).	The N-back task achieved the best accuracy (76.67%) with a mean MHbO in the interval of 5 to 25 s and an SHbO slope from 0 to the peak value, in the middle prefrontal cortex with linear discriminant analysis. Significant differences in neural biomarkers were independent of the different tasks, but the N-back task is recommended for use in early AD detection.
Nguyen et al., 2019 [43]	MCI (*n* = 42) HC matched for age. (*n* = 42)	MMSE, SNSB Specific scores were not provided.	75.9 ± 3.6 (MCI) 74.3 ± 4.4 (HC)	None/Resting state Oddball task [Attention, Processing Speed, and Working Memory] 1-Back [Working Memory] Verbal Fluency Tasks: Semantic Fluency Task and Letter Fluency Test [Verbal ability, Executive functions (Updating of Working Memory, Inhibitory control, and Cognitive flexibility)]	Significantly greater inter-hemispheric than intra-hemispheric connectivity in the HC group during the verbal fluency task—no difference between the inter- and intra-hemispheric connectivity in the MCI group. Right and inter-hemispheric connectivity was higher in MCI during the resting state. Left and inter-hemispheric connectivity was lower in MCI during verbal fluency tasks.	Functional connectivity networks may identify MCI patients at an early stage.
Yoon et al., 2019 [31]	Amnestic MCI (*n* = 9) Non-amnestic MCI (*n* = 6) HC matched for age, sex and education level (*n* = 12)	MMSE, SNSB II The study does not provide the specific scores on the clinical assessment scales.	66.9 ± 7.0 (amnestic MCI) 68.4 ± 6.5 (non-amnestic MCI) 67.8 ± 5.7 (HC)	2-Back task [Working Memory] Korean Color Word Stroop task [Executive functions (Inhibitory control and Cognitive flexibility)] Semantic Verbal Fluency Task [Verbal ability, Executive functions (Updating of Working Memory, Inhibitory control, and Cognitive flexibility)]	Right prefrontal cortex: Dominant lateralization in non-amnestic MCI and HC groups during the Stroop test. Higher activation in the non-amnestic MCI compared to the HC group during the Stroop test—hypoactivation in the amnestic MCI group.	There were active compensatory mechanisms in the right prefrontal cortex in the non-amnestic MCI group but not in the amnestic MCI group.
Kim et al., 2019 [30]	MCI (*n* = 30) HC (*n* = 39)	The study does not provide performance differences on any clinical assessment scales.	76.0 ± 3.5 (MCI) 72.2 ± 5.6 (HC)	Phonemic and Semantic Verbal Fluency Tasks [Verbal ability, Executive functions (Updating of Working Memory, Inhibitory control, and Cognitive flexibility)]	Prefrontal cortex: Higher activation in MCI than in HC.	MCI patients capitalized on compensatory mechanisms to perform the same task as the HC.
Niu et al., 2019 [44]	Amnestic MCI (aMCI) (*n* = 25) AD (*n* = 23) HC matched for age (*n* = 30)	MMSE: 23.5 ± 4.8 (aMCI), 15.5 ± 5.7 (AD), 28.2 ± 3.1 (HC) MoCA: 18.9 ± 5.4 (aMCI), 10.7 ± 5.2 (AD), 25.5 ± 3.8 (HC)	71.0 ± 8.1 (aMCI) 72.1 ± 9.3 (AD) 67.6 ± 9.0 (HC)	None/Resting state with eyes closed	Disrupted dynamic brain connectivity in aMCI and AD with increased variability over progression from HC to MCI to AD. The influence of MCI and AD was more pronounced on long-distance connections (regions of the default mode network and frontal–parietal network).	Functional connectivity networks are deregulated in MCI and AD, reducing cognitive function.
Li et al., 2018 [39]	MCI (*n* = 9) Mild AD (mAD) (*n* = 6) Moderate/severe AD (M/SAD) (*n* = 7) HC matched for age and education (*n* = 8)	MMSE: 26.0 ± 2.2 (MCI), 19.7 ± 3.0 (mAD), 9.4 ± 1.7 (M/SAD), 28.2 ± 2.2 (HC)	70.3 ± 5.4 (MCI) 72.5 ± 7.3 (mAD) 76.0 ± 4.8 (M/SAD) 63.6 ± 6.5 (HC)	Digit Verbal Span task [Short-Term Verbal Memory and Verbal Working Memory Capacity]	Frontal and bilateral parietal cortices: Increasingly reduced activation as disease severity develops from MCI to M/SAD.	Natural compensatory ability might be reduced or lost in the progression of MCI towards AD.
Katzorke et al., 2018 [40]	MCI (*n* = 55) HC matched for age, sex, years of education, Apolipoprotein-E, family history of dementia, BMI, and depression screening scores (*n* = 55)	MMST: 28.8 ± 1.3 (MCI), 29.0 ± 1.2 (HC) DemTect: 14.6 ± 2.3 (MCI), 15.8 ± 2.4 (HC) B-ADL: 1.3 ± 0.3 (MCI), 1.4 ± 0.6 (HC)	74.0 ± 1.6 (MCI) 74.2 ± 1.6 (HC)	Verbal Fluency Task [Verbal ability, Executive functions (Updating of Working Memory, Inhibitory control, and Cognitive flexibility)]	Bilateral inferior frontotemporal regions: Decreased activation for MCI patients during the category task.	Due to the role of the inferior frontotemporal cortex during the category Verbal Fluency Task, a decreased hemodynamic response of this region could serve as a biomarker for the diagnosis of MCI in the future.
Yap et al., 2017 [34]	MCI (*n* = 12) Mild AD (*n* = 18) HC matched for age, gender and education (*n* = 31)	MMSE: 26.0 ± 3.1 (MCI), 21.2 ± 3.6 (mild AD), 28.7 ± 1.5 (HC) CDR: 0.5 (MCI), 1.0 (mild AD), 0.0 (HC)	73.1 ± 8.2 (MCI) 74.7 ± 10.0 (mild AD) 72.6 ± 8.5 (HC)	Semantic Verbal Fluency Task [Verbal ability, Executive functions (Updating of Working Memory, Inhibitory control, and Cognitive flexibility)]	Prefrontal cortex, right and left: Although statistically insignificant, MCI had a greater average activation than HC and AD. Left prefrontal cortex: Shorter time to achieve activation in HC than MCI-AD. Right prefrontal cortex: Longer time to achieve activation in AD than HC-MCI.	Natural compensatory ability might be reduced or lost in the progression of MCI towards AD. The lower activation and longer time needed for prefrontal cortex activation in AD might suggest that the compensatory mechanism is compromised. The more pronounced activation in MCI is possibly a compensatory response.
Yeung et al., 2016 [36]	MCI (*n* = 26) HC matched for age, gender, handedness, and education (*n* = 26)	CDRS: 149.8 ± 6.8 (MCI), 153.4 ± 6.3 (HC)	69.2 ± 6.3 (MCI) 68.9 ± 6.1 (HC)	0-, 2-Back task [Working Memory (WM)]	Bilateral frontal and frontopolar regions: Activation in HC during high working memory load. The MCI group exhibited similar activation at low load but reduced activation with increased load compared to the HC group.	The absence of task-related activation in the MCI group might be attributable to their failure to deploy compensatory effort in response to increasing task demands.
Uemura et al., 2016 [41]	Amnestic MCI (aMCI) (*n*= 64) HC matched for age and gender (*n* = 66)	MMSE: 26.7 ± 1.8 (aMCI), 27.7 ± 1.6 (HC)	71.8 ± 4.3 (aMCI) 71.7 ± 3.9 (HC)	Memory encoding and delayed retrieval of ten target words [Episodic and/or Semantic Memory processing]	Bilateral dorsolateral prefrontal cortex: Reduced activation in MCI during the memory retrieval task only.	MCI patients failed to recruit sufficient prefrontal resources for the retrieval memory task and did not show the expected task-related activation exhibited by the control group.
Niu et al., 2013 [37]	MCI (*n* = 8) HC matched for age and gender (*n* = 16)	MMSE: 26.3 ± 2.3 (MCI), 28.4 ± 1.1 (HC)	64.8 ± 7.2 (MCI) 63.1 ± 5.3 (HC)	0-, 1-Back task [Working Memory]	Left dorsolateral prefrontal, right supplementary motor, and left superior temporal regions: Decreased activation in the MCI group.	MCI patients failed to recruit sufficient frontotemporal resources for the task and did not show the expected task-related activation exhibited by the control group.
Arai et al., 2006 [42]	Probable AD (*n* = 15) Amnestic MCI (aMCI) (*n* = 15) HC matched for age, sex, and education (*n* = 32)	MMSE: 15.1 ± 7.0 (AD), 26.3 ± 1.6 (aMCI), 29.1 ± 0.8 (HC)	59.2 ± 3.9 (AD) 63.0 ± 6.4 (aMCI) 57.3 ± 6.4 (HC)	Verbal Fluency Task [Verbal ability, Executive functions (Updating of Working Memory, Inhibitory control, and Cognitive flexibility)]	Bilateral frontal and parietal lobes: Lower activation in the AD group than in the HC group. Right parietal area: Lower activation in the MCI group than in the HC, with activation index in the middle between those of the HC and AD groups.	The hypoactivation in the AD group during the verbal task was relatively global and differs from that of healthy controls and those with MCI.

Note: Quantitative data are presented as the mean ± standard deviation; fNIRS: functional near-infrared spectroscopy; AD: Alzheimer’s disease; MCI: mild cognitive impairment; HC: healthy controls; (K-) MMSE: (Korean-) Mini Mental State Examination; MoCA: Montreal Cognitive Assessment; DemTect: Dementia Detection Test; B-ADL: Bayer Activities of Daily Living Scale; CDR: Clinical Dementia Rating; SNSB: Seoul Neuropsychological Screening Battery; MMST: Mini Mental Status Test; CDRS: Chinese Version of the Mattis Dementia Rating Scale.

### 3.2. fNIRS in AD

AD is the most prevalent neurodegenerative disorder, a leading cause of death and healthcare burden; the aging of the global population and the improvement of—and increased access to—healthcare services are expected to cause the prevalence and incidence of this major neurocognitive entity to skyrocket [46,47,48]. AD is usually characterized by early prominent episodic memory impairment along with more subtle cognitive deficits in the remaining cognitive domains and a variety of neuropsychiatric manifestations, such as affective and lability symptoms, apathy, and even psychotic manifestations [25,49]. The ATN [β amyloid, tau, neurodegeneration] framework has been introduced to define the underlying neurodegenerative alterations of the disorder and tends to displace the traditional clinical diagnostic approach; β amyloid deposition, tau aggregation, and neurodegenerative changes characteristic of AD not only improve its challenging differential diagnosis but also facilitate early identification—even in a preclinical stage—allowing timely interventions and serving research purposes [50,51,52].

The literature search yielded 12 relevant articles (Table 2). Studies comparing participants with AD to HC uniformly report findings of cortical hypoactivation in the former group. Herrmann and colleagues found reduced (dorsolateral prefrontal cortex) and less locally specific activation during the verbal fluency task [53]. Zeller and colleagues documented lower activation of the superior parietal cortex during the modified version of the Benton Line Orientation Task [54]. Metzger and colleagues showed hypoactivation of frontoparietal areas (such as the dorsolateral prefrontal cortex) and the superior temporal gyrus during the verbal fluency task [55]. Li and colleagues reported that patients assessed on the digit verbal span task presented lower activation of frontal regions (frontal pole, orbitofrontal) [56]. Arai and colleagues found lower activation of the bilateral frontal and parietal lobes of those with AD during the verbal fluency task [42]. Li and colleagues revealed increasingly reduced activation of frontal and bilateral parietal cortices in the course of progression from mild to moderate/severe AD during the digit verbal span task [39]. Yap and colleagues observed lower and relatively delayed activation of the left prefrontal cortex during the verbal fluency task [34]. Ung and colleagues showed less pronounced bilateral prefrontal activation with minimal signs of increasing activation with increasing difficulty level during a visuospatial working memory task [29]. Based on the above, it can be theorized that compensatory mechanisms may exist early in the course of neurodegeneration (early MCI) but are compromised later on (late MCI, dementia stage). MCI patients may be able to handle increasing cognitive load using compensatory mechanisms at first until they reach their cognitive capacity limits for neural compensation due to more severe neurodegeneration. 

Alternative parameters were assessed in a number of articles. Niu and colleagues evaluated participants with AD, amnestic MCI, and HC in terms of functional brain connectivity during a resting state [44]. The authors found increasingly disrupted dynamic brain connectivity with escalating variability over progression from normal cognition to MCI and AD. Perpetuini and colleagues analyzed the complexity of activation based on multiscale entropy metrics [57]. Those with mild AD exhibited increased complexity of activation during delayed free recall in the dorsolateral and medial prefrontal cortex but comparable complexity to that of the HC during the resting state and other episodic memory tasks. Ateş and colleagues revealed that patients with AD may show relative preservation of working memory performance when positive emotional stimuli are used in contrast to the use of neutral or negative emotional stimuli [58]. This function was associated with higher activation of the left ventral prefrontal cortex in patients with AD during the positive condition (and not during neutral and negative conditions). Therefore, positive verbal stimuli were suggested to enhance working memory performance among older adults with AD. Finally, two published articles highlighted the potential of combining fNIRS with other modalities—specifically, EEG [56,59]. Multimodal evaluation of neurovascular coupling is even more promising in the identification of undergoing neurodegenerative alterations.

**Table 2:** fNIRS studies involving older adults with AD and HC.

Study	Participants	Clinical Scores	Mean Age in Years ± SD	Tasks [Corresponding Functions]	fNIRS Data [Mentioned Involved Areas]	Conclusions
Chiarelli et al., 2021 [59]	Mild AD (*n* = 17) HC matched for gender, age, and education (*n* = 18)	MMSE: 22.9 ± 3.2 (AD), 27.5 ± 2.2 (HC)	75.1 ± 7.1 (AD) 71.4 ± 7.8 (HC)	None/Resting state with eyes closed	Standalone fNIRS metrics did not highlight differences between AD and HC.	Unimodal evaluation of global hemodynamic brain activity with fNIRS did not highlight statistically significant differences between AD and HC. Multimodal analysis (combined with EEG) is more likely to reveal neurovascular coupling alterations and assist in the early identification of AD.
Ung et al., 2020 [29]	MCI (*n* = 12) Mild AD (mAD) (*n* = 18) HC matched for age, gender and educational level. (*n* = 31)	MMSE: 26.0 ± 3.1 (MCI), 21.2 ± 3.6 (mAD), 28.7 ± 1.5 (HC) CDR: 0.5 (MCI), 1.0 (mAD), 0.0 (HC)	73.1 ± 8.2 (MCI) 74.7 ± 10.0 (mAD) 72.6 ± 8.5 (HC)	Visuospatial working memory task based on “Neuro Recall” [Visuospatial Working Memory]	Bilateral prefrontal cortex: Increasing activation with increasing task difficulty in HC and MCI—even steeper increase in MCI. Little sign of increasing activation as task difficulty increases in AD.	MCI patients could handle a low visuospatial working memory load without the need to compensate. At higher levels of difficulty, compensatory mechanisms were recruited. AD patients could not recruit any compensatory mechanisms.
Li et al., 2019 [56]	Mild AD (mAD) (*n* = 6) HC matched for age and gender (*n* = 8)	MMSE: 19.7 ± 3.0 (mAD), 28.1 ± 1.1 (HC)	72.50 ± 7.34 (mAD) 62.75 ± 8.21 (HC)	Digit Verbal Span task [Short-Term Verbal Memory and Verbal Working Memory capacity]	Frontal regions—frontal pole, orbitofrontal: Similar activation patterns between the two groups, with lower activation in AD. Left hemisphere, especially the left frontal pole and orbitofrontal cortices: Lower functional connectivity in interhemispheric connections in AD.	These findings could serve as ‘‘network biomarkers’’ in the early identification of AD. EEG integration might contribute to the better understanding of spatiotemporal brain dynamics and increase the discriminative properties of unimodal fNIRS assessments.
Niu et al., 2019 [44]	Amnestic MCI (aMCI) (*n* = 25) AD (*n* = 23) HC matched for age (*n* = 30)	MMSE: 23.5 ± 4.8 (aMCI), 15.5 ± 5.7 (AD), 28.2 ± 3.1 (HC) MoCA: 18.9 ± 5.4 (aMCI), 10.7 ± 5.2 (AD), 25.5 ± 3.8 (HC)	71.0 ± 8.1 (aMCI) 72.1 ± 9.3 (AD) 67.6 ± 9.0 (HC)	None/Resting state with eyes closed	Disrupted dynamic brain connectivity in aMCI and AD with increased variability over progression from HC to MCI to AD. The influence of MCI and AD was more pronounced on long-distance connections (regions of the default mode network and frontal–parietal network).	Functional connectivity networks are deregulated in MCI and AD, reducing cognitive function.
Li et al., 2018 [39]	MCI (*n* = 9) Mild AD (mAD) (*n* = 6) Moderate/severe AD (M/SAD) (*n* = 7) HC matched for age and education (*n* = 8)	MMSE: 26.0 ± 2.2 (MCI), 19.7 ± 3.0 (mAD), 9.4 ± 1.7 (M/SAD), 28.2 ± 2.2 (HC)	70.3 ± 5.4 (MCI) 72.5 ± 7.3 (mAD) 76.0 ± 4.8 (M/SAD) 63.6 ± 6.5 (HC)	Digit Verbal Span task [Short-Term Verbal Memory and Verbal Working Memory capacity]	Frontal and bilateral parietal cortices: Increasingly reduced activation as disease severity develops from MCI to M/SAD.	Natural compensatory ability might be reduced or lost in the progression of MCI towards AD.
Yap et al., 2017 [34]	MCI (*n* = 12) Mild AD (*n* = 18) HC matched for age, gender and education (*n* = 31)	MMSE: 26.0 ± 3.1 (MCI), 21.2 ± 3.6 (mild AD), 28.7 ± 1.5 (HC) CDR: 0.5 (MCI), 1.0 (mild AD), 0.0 (HC)	73.1 ± 8.2 (MCI) 74.7 ± 10.0 (mild AD) 72.6 ± 8.5 (HC)	Semantic Verbal Fluency Task [Verbal ability, Executive functions (Updating of Working Memory, Inhibitory control, and Cognitive flexibility)]	Prefrontal cortex, right and left: Although statistically insignificant, MCI had a greater average activation than HC and AD. Left prefrontal cortex: Shorter time to achieve activation in HC than in MCI–AD. Right prefrontal cortex: Longer time to achieve activation in AD than in HC–MCI.	Natural compensatory ability might be reduced or lost in the progression of MCI towards AD. The lower activation and longer time needed for prefrontal cortex activation in AD might suggest that the compensatory mechanism is compromised. The more pronounced activation in MCI is possibly a compensatory response.
Ateş et al., 2017 [58]	AD (*n* = 20) HC differed significantly with respect to age and total years of education (*n* = 20)	MMSE: 18.6 ± 5.0 (AD), 26.5 ± 1.8 (HC) FAQ: 13.5 ± 9.0 (AD), 1.4 ± 3.4 (HC)	76.3 ± 5.2 (AD) 71.4 ± 6.8 (HC)	Verbal Emotional n-Back Task (positive, negative, and neutral condition) [Emotional processing, Working Memory]	Left ventral prefrontal cortex: Higher activation in the AD group only for the positive condition (not for negative or neutral). AD performed worse than HC in neutral and negative but not in positive word conditions.	The results of the present study suggest that patients with AD show a relative preservation of working memory performance when positive emotional stimuli are used. Higher left-sided activity in AD patients could reflect an enhancement effect of positive verbal stimuli.
Perpetuini et al., 2017 [57]	Mild AD (*n* = 11) HC (*n* = 11)	Without moderate–severe cognitive impairment (Mini Mental State Examination >24/30).	72.2 ± 4.5 (AD) 67.5 ± 5.0 (HC)	Free and Cued Selective Reminding Task [Verbal episodic memory with controlled learning and semantic cueing] None/Resting state	Dorsolateral and medial prefrontal cortex: Increased complexity of activation (based on multiscale entropy metrics) in the AD group during delayed free recall. No significant difference in between groups’ sample entropy during the resting state.	The higher complexity of the AD group could be a result of a dysfunction in the neurovascular coupling in the frontal area.
Metzger et al., 2016 [55]	Behavioral variant of FTD (bvFTD) (*n* = 8) AD matched for age, gender, education, and behavioral data in the Verbal Fluency Task with the bvFTD group (*n* = 8) HC matched for gender, age, education, and medication with the bvFTD group (*n* = 8)	CERAD- Plus test battery. The study does not provide the specific scores on the clinical assessment scales.	67.6 ± 9.8 (bvFTD) 74.3 ± 4.5 (AD) 65.5 ± 6.5 (HC)	Verbal Fluency Task [Verbal ability, Executive functions (Updating of Working Memory, Inhibitory control, and Cognitive flexibility)]	Frontoparietal areas such as the dorsolateral prefrontal cortex—superior temporal gyrus: This pattern of activation was revealed in the HC. Participants with AD had an activation pattern similar to but weaker than that of the HC. Frontopolar areas—Broca’s area: This pattern of activation was revealed in bvFTD.	Compared to HC, compensatory ability might be reduced or lost in AD. bvFTD pattern is qualitatively different, namely, more frontopolar and without frontoparietal compensation activation.
Zeller et al., 2010 [54]	Mild AD (mAD) (*n* = 13) HC matched for age and gender (*n* = 13)	MMSE: 23.0 ± 3.37 DemTect 10.6 ± 5.17	61.7 ± 6.2 (mAD) 61.8 ± 5.5 (HC)	Modified version of the Benton Line Orientation Task [Visuospatial ability]	Superior parietal cortex: Higher activation in the HC and less pronounced activation in mAD, although visuospatial task performance was similar.	Neurofunctional deficits may precede neuronal degeneration, underlining the use of fNIRS as a potential diagnostic tool in early stages.
Herrmann et al., 2008 [53]	Mild to moderate AD (*n* = 16) HC matched for age and sex (*n* = 16)	MMSE: 19.9 ± 4.6 (AD) Disease duration: 2.06 ± 4.6 DemTec: 6.1 ± 3.0 (AD), 16.4 ± 1.7 (HC)	69.5 ± 8.4 (AD) 67.9 ± 5.4 (HC)	Verbal Fluency Task [Verbal ability, Executive functions (Updating of Working Memory, Inhibitory control, and Cognitive flexibility)]	Dorsolateral prefrontal cortex: Reduced activation and less locally specific activation pattern in the AD group compared to the HC (for both letter and category tasks, but more pronounced for letter tasks).	The less pronounced activation in association with no locally specific activation pattern in AD opposes the idea of a compensatory prefrontal network in AD.
Arai et al., 2006 [42]	Probable AD (*n* = 15) Amnestic MCI (aMCI) (*n* = 15) HC matched for age, sex and education (*n* = 32)	MMSE: 15.1 ± 7.0 (AD), 26.3 ± 1.6 (aMCI), 29.1 ± 0.8 (HC)	59.2 ± 3.9 (AD) 63.0 ± 6.4 (aMCI) 57.3 ± 6.4 (HC)	Verbal Fluency Task [Verbal ability, Executive functions (Updating of Working Memory, Inhibitory control, and Cognitive flexibility)]	Bilateral frontal and parietal lobes: Lower activation in the AD group compared to the HC group. Right parietal area: Lower activation in the MCI group compared to the HC, with the activation index being in the middle between those of the HC and AD groups.	The hypoactivation in the AD group during the verbal task was relatively global and differed from that of healthy controls and those with MCI.

Note: Quantitative data are presented as the mean ± standard deviation; fNIRS: functional near-infrared spectroscopy; AD: Alzheimer’s disease; HC: healthy controls; MCI: mild cognitive impairment; CDR: Clinical Dementia Rating; MMSE: Mini Mental State Examination; MoCA: Montreal Cognitive Assessment; FAQ: Functional Activities Questionnaire; CERAD: Consortium to Establish a Registry for Alzheimer’s Disease.; DemTec: Dementia Detection test.

### 3.3. fNIRS in FTD

Frontotemporal dementia (FTD) is a major neurocognitive disorder with two common phenotypic presentations: primary progressive aphasia (PPA) with early prominent language impairment and the behavioral variant (bvFTD) with early alterations in emotion, personality, and executive function [60,61]. Frontal and/or anterior temporal atrophy in magnetic resonance imaging (MRI) studies or hypometabolism in fluro-deoxy-glucose positron emission tomography (FDG-PET) are imaging markers of bvFTD [62,63]. Apart from these presentations, additional entities on the spectrum include FTD with motor neuron disease (MND), corticobasal degeneration (CBD), and progressive supranuclear palsy (PSP) [61].

Only one relevant article involving HC, individuals with AD, and the behavioral variant of FTD (bvFTD) was retrieved [55] (Table 2). As mentioned above, compared to the HC, the compensatory ability during the verbal fluency task was reduced in AD; however, the pattern of cortical activation (though less pronounced) was similar to that of the HC (frontoparietal areas such as the dorsolateral prefrontal cortex—superior temporal gyrus). On the other hand, the bvFTD pattern was qualitatively different, namely, more frontopolar—without frontoparietal activation. This study provides evidence that compensatory mechanisms may differ between different neurodegenerative diseases. This is an indication of the diverse neuropathophysiological correlates that can be capitalized upon in the challenging distinction of these conditions. 

### 3.4. fNIRS in PD

PD is a progressive neurodegenerative disorder of the central nervous system (CNS) marked by cardinal movement manifestations involving resting tremor, rigidity, bradykinesia, and postural instability [64]. Autonomic dysfunction, anosmia, and sleep, cognitive, and neuropsychiatric symptoms may occur. PD is associated with the degeneration of dopamine-producing neurons in the pars compacta of the substantia nigra [65]. Cytoplasmic inclusions of a-Synuclein-forming Lewy bodies and neurites tend to accumulate within affected neurons [65,66,67]. Following AD, it is the most common neurodegenerative disorder, as well as the most prevalent entity among aS-pathies [66].

The analysis of fNIRS data in PD versus HC provides evidence of the capitalization of cognitive resources (especially executive function) for the compensation of locomotor impairments (Table 3). Ranchet and colleagues examined early-stage PD patients and HC during simple and dual walking tasks [68]. They found higher activation of the dorsolateral prefrontal cortex during usual walking and during walking while subtracting in PD, supporting that prefrontal activation is potentially compensatory for subcortical dysfunction and deficits in motor automaticity. Shine and colleagues assessed early-stage PD patients and HC using the obstacle negotiation task [69]. In their study, greater activation of the prefrontal region was exhibited during and after this task in PD, especially in the case of more challenging obstacles. These results point to the reliance of patients with PD on cognitive resources during more demanding motor situations. Mahoney and colleagues examined patients with Parkinsonian syndromes, individuals with mild Parkinsonian signs, and HC during the postural control task [70]. Their findings support that increasing activation of the prefrontal cortex is required in Parkinsonian syndromes in order to retain postural control compared to those with mild Parkinsonian signs and HC. Belluscio and colleagues evaluated PD patients with (PD-FoG) and without freezing of gait (PD-no FoG) and HC on the 2-min turning-in-place task under single-task and dual-task conditions [71]. The authors reported higher activation of the prefrontal cortex in the PD-FoG group. They theorized that the involvement of the prefrontal cortex during a challenging motor task in PD implies the increasing need for the recruitment of executive mechanisms in motor tasks with declining motor performance in the course of PD. Pu and colleagues assessed participants with PD-noFOG, PD-FoG, and HC on the sitting toe-tapping task [72]. The greater right prefrontal activation in the PD-FoG group once again suggested that PD-FoG patients require additional cognitive resources to compensate for damaged automaticity in locomotor control. This is more pronounced in cases with more severe FoG than in milder cases.

Additional evidence on the implication of frontally mediated operations (most notably, executive function) in the locomotor performance of patients with PD was provided by the studies of Maidan and colleagues. Individuals with PD-FoG and HC were subjected to different walking tasks known to provoke FoG [73]. Increased frontal activation was found before and during anticipated (but not unanticipated) turns with FoG. Later, Maidan and colleagues assessed individuals with PD and HC on the obstacle negotiation task [74]. Higher prefrontal activation was found in PD before, during, and after the task in both anticipated and unanticipated obstacle negotiation. Finally, Maidan and colleagues investigated older adults with PD and HC on usual walking, dual walking, and obstacle negotiation tasks [75]. Higher activation of the prefrontal cortex was reported during the usual walking and obstacle negotiation tasks. The activation was similar to that in the HC during the dual walking tasks. The authors pointed out that pure motor tasks led to increased frontal lobe activation only in the PD group (neural compensation), whereas cognitive operations during dual walking apparently led to increased frontal lobe activation in the HC group as well. 

On the other hand, data indicative of the failure of compensatory mechanisms in PD have also been published. Pelicioni and colleagues examined patients with mild to moderate PD and HC on simple walking and three gait adaptability tasks [76]. The authors observed that the PD group had greater activation of the premotor cortex during simple walking (compared to the HC) but no increasing activation with escalating task difficulty in the dorsolateral prefrontal cortex (as seen in the HC). Their findings may suggest that people with PD have little premotor cortex and dorsolateral prefrontal cortex capacity beyond what they need for simple walking. A second study by Pelicioni and colleagues evaluated mild to moderate PD patients and HC on the simple choice stepping reaction time task, the inhibitory choice stepping reaction time task, and the Stroop stepping task [77]. Reduced activation of the dorsolateral prefrontal cortex, supplementary motor area, and premotor cortex was found during more complex tasks requiring inhibitory control. This finding may reflect subcortical damage with subsequent deficient use of compensatory cognitive and motor resources. Overall, similarly to neurocognitive entities, depending on the severity and exact phenotype of PD (motor, cognitive, neuropsychiatric manifestations, and so on), as well as on the exact demands of the evaluations utilized, heterogeneity is to be expected. Future research ought to tackle these issues by forming more clinically homogeneous groups.

Finally, one article by Hofmann and colleagues assessed PD converters (almost every one of whom was not diagnosed with PD at the time of the examination) and HC using the Trail-Making Test [78]. They found reduced activation of the right dorsolateral prefrontal cortex with increasing task difficulty in PD—on the contrary, the HC exhibited increasing activation with escalating task difficulty. Regarding the left dorsolateral prefrontal cortex, increasing activation with escalating task difficulty was reported in both groups. This could be an early and presumably PD-specific pattern of cortical activation.

**Table 3:** fNIRS studies involving older adults with PD and HC.

Study	Participants	Clinical Scores	Mean Age in Years ± SD	Tasks [Corresponding Functions]	fNIRS Data—Non-Resting State	Conclusions
Pelicioni et al., 2022 [76]	Mild to moderate PD (*n* = 49) HC (*n* = 21)	MMSE: 28.8 ± 1.3 (PD), 29.2 ± 1.1 (HC) Disease duration: 6.8 ± 4.8 years UPDRS-II: 12.8 ± 6.4 UPDRS-III: 31.6 ± 10.1 UPDRS-IV: 3.0 ± 3.6 Hoehn and Yahr stage: 74% II, 22% III, 4% I NFG-Q: 5.4 ± 8.2	69.5 ± 7.9 (PD) 69.0 ± 5.9 (HC)	Simple walking and gait adaptability; (i) stepping on targets, (ii) avoiding obstacles, and (iii) negotiating both targets and obstacles [Motor functions such as balance, stability, adaptability to varying conditions, motor planning, inhibitory control, and decision making]	Premotor cortex: Greater activation in PD during simple walking compared to the HC. Dorsolateral prefrontal cortex: No increasing activation with increasing task difficulty in PD, unlike in the HC.	People with PD do not increase their cortical activity levels when undertaking complex gait adaptability tasks requiring inhibitory control. They have little to no premotor cortex and dorsolateral prefrontal cortex capacity beyond what they need for simple walking.
Pu et al., 2022 [72]	PD no-freezing of gait (PD-no FoG) (*n* = 19) PD-FoG) (*n* = 37) HC matched for age (*n* = 34)	MMSE: 27 ± 2 (PD-no FoG), 26 ± 2 (PD-FoG), 28 ± 2 (HC) UPDRS-III: 26.0 ± 9.2 (PD-no FoG), 40.2 ± 16.7 (PD-FoG) FG-Q: 12.4 ± 4.4 (PD-FoG)	63 ± 9 (PD no- FoG) 67 ± 9 (PD-FoG) 65 ± 7 (HC)	Sitting toe-tapping task [Lower-extremity/ lower-limb locomotor functions]	Right prefrontal cortex: Greater activation in the PD-FoG group than in the other two groups	PD-FoG requires additional cognitive resources to compensate the damaged automaticity in locomotor control. This is more pronounced in cases with more severe FoG than in milder cases.
Hofmann et al., 2021 [78]	PD-converters [Early PD-converters (*n* = 9), Late PD-converters (*n* = 12)] HC (*n* = 21) matched for age, gender, and years of education	CERADplus battery, MOCA. The study does not provide the specific scores on assessment scales.	71.9 ± 4.6 (PD-converters) 72 ± 4.7 (HC)	Trail-making test: TMT-C (control condition), TMT-A (simple condition), TMT-B (complex condition) [Processing speed, Sequencing, Cognitive flexibility, and Visual–motor skills]	Left dorsolateral prefrontal cortex: Increasing activation with increasing task difficulty in the HC and PD. Right dorsolateral prefrontal cortex: Increasing activation with increasing task difficulty in the HC. Right dorsolateral prefrontal cortex and sensory association cortex: Generally reduced activation during tasks in PD.	A higher cortical activity due to the more complex task is preserved even within the group of PD converters in the left dorsolateral prefrontal cortex. Reduced activation of the right dorsolateral prefrontal cortex despite escalating task difficulty could be an early and presumably specific pattern for conversion into PD.
Pelicioni et al., 2020 [77]	Mild to moderate PD (*n* = 52) HC matched for age (*n* = 95)	MMSE: 28.8 ± 1.3 (PD), 29.2 ± 0.9 (HC) UPDRS-II: 12.6 ± 6.3 UPDRS-III: 31.6 ± 10.4 Hoehn and Yahr stage: 73% II, 23% III, 4% I	70.2 ± 8.4 (PD) 71.3 ± 4.9 (HC)	Simple choice stepping reaction time task [Balance, mobility, and reaction time] Inhibitory choice stepping reaction time task [Perceptual and motor inhibitory control] Stroop stepping task [Balance, mobility, and inhibitory control]	Dorsolateral prefrontal cortex, supplementary motor area, premotor cortex: Reduced activation during more complex tasks requiring inhibitory control in PD.	The reduced activation may reflect subcortical and/or multiple-pathway damage with subsequent deficient use of cognitive and motor resources.
Ranchet et al., 2020 [68]	Early-stage PD (*n* = 18) HC (*n* = 18)	MoCA 27.5 (26.0–29.0) (PD), 27.0 (26.0–29.0) (HC) UPDRS: 17 (12–26) Hoehn and Yahr stage: 2 (2–2) BDI: 6 ± 4 (PD), 2 ± 2 (HC) FES-I: 25 ± 8 (PD), 19 ± 3 (HC)	68 ± 8 (PD) 66 ± 7 (HC)	Standing while subtracting [Standing: motor function, posture maintenance ability, balance; subtracting: working memory, executive functions] Usual walking [Motor automaticity and basic motor functions (gait speed, stride time, and stride time variability)] Walking while counting forward [Walking: automaticity and basic motor functions (gait speed, stride time, and stride time variability); counting forward: working memory and short-term memory], Walking while subtracting [Walking: automaticity and basic motor functions (gait speed, stride time, and stride time variability); subtracting: working memory, executive functions]	Dorsolateral prefrontal cortex: Higher activation during both usual walking and walking while subtracting in PD compared to the HC.	The Increased dorsolateral prefrontal cortex activity in patients during usual walking suggests a potential compensation for motor deficits.
Shine et al., 2020 [69]	Early-stage PD (*n* = 34) HC (*n* = 26)	MoCA: 27.0 [20.0, 30.0] (PD), 26.5 [19.0, 30.0] (HC) Disease duration: 3.5 [0.4, 15.0] years UPDRS-ΙΙΙ score: 27.0 [5.0, 50.0] Hoehn and Yahr stage: 2 [1, 3]	67.4 ± 5.7 (PD) 71.3 ± 8.9 (HC)	Obstacle negotiation task [Motor control (gait stability and adaptability)]	Prefrontal region: Greater activation during and after the task in PD compared to the HC, especially in the case of more challenging obstacles.	These results point to the use of prefrontal activation as a compensatory mechanism in PD. There is a greater reliance on cognitive resources in more demanding motor situations in patients with PD.
Belluscio et al., 2019 [71]	PD-no freezing of gait (PD-no FOG) (*n* = 17) PD-FoG (*n* = 15) HC matched for age (*n* = 8)	MoCA: 25.4 ± 3.8 (PD-no FoG), 28.7 ± 1.3 (PD-FoG), 26.6 ± 1.9 (HC) Disease duration: 9.35 ± 6.7 years (PD-no FoG), 13.5 ± 6.0 years (PD-FoG) UPDRS-III: 33.5 ± 11.2 (PD-no FoG), 46.9 ± 11.8 (PD-FoG)	69.9 ± 4.3 (PD-no FoG) 66.9 ± 5.0 (PD-FoG) 66.5 ± 5.5 (HC)	2-min turning-in-place task as a single task and dual task (additional execution of an auditory modified AX-continuous performance task) [Turning-in-place test: coordination, balance, postural control, spatial awareness, motor planning; auditory modified AX-continuous performance task: additional auditory and visual context-dependent information processing/contextual working memory and cognitive control]	Prefrontal cortex: Higher activation during the task in PD-FoG compared to the other groups. Higher activity is related to worse FoG and a lower number of turns.	Involvement of the prefrontal cortex in people with PD while performing a challenging task may imply the recruitment of executive function in performing a motor task among individuals with poorer motor performance.
Maidan et al., 2019 [74]	PD (*n* = 34) HC (*n* = 26)	The conference abstract does not provide the specific scores on the clinical assessment scales.	67.4 ± 5.7 (PD) 71.3 ± 8.9 (HC)	Obstacle negotiation task [Motor control, particularly gait stability and adaptability]	Prefrontal cortex: Higher activation in PD during task in both anticipated and unanticipated obstacle negotiation.	The prefrontal cortex has a role during both anticipated and unanticipated obstacle negotiation in PD.
Mahoney et al., 2016 [70]	Parkinsonian syndromes (PSs) (*n* = 26) Mild Parkinsonian Signs (MPSs) (*n* = 117) HC (*n* = 126)	RBANS: 91.04 ± 11.12 (PS), 89.82 ± 12.85 (MPS), 93.75 ± 11.10 (HC) MPS severity score: 11.08 ± 3.60 (PS), 3.21 ± 2.49 (MPS) GDS: 6.15 ± 3.88 (PS), 4.90 ± 4.04 (MPS), 4.12 ± 3.14 (HC)	81.23 ± 5.93 (PS) 77.50 ± 6.72 (MPS) 74.41 ± 6.12 (HC)	Postural control task [Motor functions (coordination and balance)]	Prefrontal cortex: Increasing activation in PD in order to complete the task compared to MPSs and the HC.	The increased activation in the prefrontal cortex required by PD highlights the role of this region in postural control in patients with PSs.
Maidan et al., 2016 [75]	PD (*n* = 68) HC (*n* = 38)	MMSE: 28.8 ± 0.2 (PD), 28.2 ± 0.2 (HC) Disease duration: 9.35 ± 6.7 years UPDRS-III: 9.1 ± 0.7	71.7 ± 1.1 (PD) 70.4 ± 0.9 (HC)	Usual walking [Automaticity and basic motor functions such as gait speed, stride time, and stride time variability] Walking while serially subtracting 3 s from a given three-digit number—dual task [Walking: automaticity and basic motor functions (gait speed, stride time, and stride time variability); subtracting: working memory, executive functions] Walking while negotiating obstacles [Walking: automaticity and basic motor functions (gait speed, stride time, and stride time variability); obstacle negotiation: [Motor control, particularly gait stability and adaptability]	Prefrontal cortex: Higher activation in PD compared to HC during usual walking and obstacle negotiation, but not during dual task walking	In healthy older adults, a cognitive task apparently leads to increased frontal lobe activation, while obstacle negotiation does so to a much lesser extent. The opposite is true in patients with PD (recruitment of prefrontal resources is greater during walking and obstacle negotiation).
Maidan et al., 2015 [73]	PD-freezing of gait (PD-FoG) (*n* = 11) HC (*n* = 11)	MoCA ≥ 24 for all participants UPDRS-III: 42.8 ± 9.3 Hoehn and Yahr stage: 2.8 ± 0.4 NFG-Q: 23.2 ± 5.3	66.2 ± 10.0 (PD-FoG) 71.2 ± 6.0 (HC)	Different walking tasks known to provoke FoG, such as performing anticipated and unanticipated turns [Motor planning, information processing, and executive control]	Frontal lobe: Increased activation before and during anticipated turns with FoG. No changes before and during unanticipated turns with FoG. Decreased activation during turns without FoG. No changes in the HC.	These associations highlight the connections among motor planning, information processing, executive control, and FoG.

Note: Quantitative data are presented as the mean ± standard deviation or median [minimum, maximum] or median (Q1–Q3, that is, the interquartile range) depending on the data presentation in the respective article; fNIRS: functional near-infrared spectroscopy, PD: Parkinson’s disease; HC: healthy controls; MMSE: Mini Mental State Examination; MoCA: Montreal Cognitive Assessment; CERAD: Consortium to Establish a Registry for Alzheimer’s Disease; RBANS: Repeatable Battery for the Assessment of Neuropsychological Status; TMT: Trail-Making Test; UPDRS: Unified Parkinson’s Disease Rating Scale; GDS: Geriatric Depression Scale; BDI: Beck Depression Inventory; FES-I: Falls Efficacy Scale—International; (N)FG-Q: (New) Freezing of Gait Questionnaire.

### 3.5. fNIRS in ALS

ALS is a progressive neurodegenerative disorder that mainly affects the upper and lower motor neurons, and about half of the patients present cognitive decline during the course of the disease [79]. The worldwide prevalence of ALS is estimated at approximately between four and five patients per 100,000 individuals, whereas its incidence corresponds to about one to two new cases per 100,000 person-years [80]. ALS is more common among males, and its prevalence follows an upward trend towards the eighth decade of life [80]. The mean survival of ALS patients is estimated between 2 and 4 years for most populations, with the limited available therapeutic options offering only small benefits in terms of survival and clinical progress [80,81,82]. 

fNIRS data from studies including ALS patients and HC support the involvement of extra-motor networks and hubs in ALS, even in the absence of measurable cognitive impairment (Table 4). Deligani and colleagues found increased functional connectivity in the frontal and right prefrontal regions of the ALS group during the resting state [83]. As activity related to constant monitoring for upcoming stimuli has been reported to occur in the resting state, increased connectivity was theorized to be a compensatory mechanism for monitoring deficits. Borgheai and colleagues assessed participants with ALS and HC (matched for age) on an oddball-based dual visual–mental task [84]. Significant hemodynamic contrast was observed primarily in the dorsolateral prefrontal cortex (a region critical to working memory processing) of the ALS group. This strengthens speculation that participants with ALS may have extra-motor impairment with prominent attentional and executive deficits affecting workload processing. Ayaz and colleagues evaluated ALS patients and HC on mental tasks targeting attention, executive function, and processing speed [85]. The ALS group had higher activation of the lateral and medial prefrontal cortex, as well as the inferior frontal gyrus, during these tasks. These significant differences between ALS and HC in fNIRS measures during all tasks provide an additional metric for the assessment of cognitive decline in these patients as well. In ALS, the activation level was highest at the beginning of a task and decreased with subsequent trials of increasing difficulty; on the contrary, an increase in activation with escalating task difficulty was reported in the HC. The authors suggested that this finding should be attributed to a higher neural cost of task initiation in ALS, while increasing the task’s difficulty exceeds the compensatory capabilities in this population. 

Kopitzki and colleagues observed no significant difference in homotopic resting-state functional connectivity (rs-FC) between ALS patients and HC [86]. However, individuals with ALS displayed an altered correlation between homotopic rs-FC values obtained at different cortical sites when compared to the HC. The altered spatial pattern of correlation in homotopic rs-FC values measured in different non-motor-associated cortical areas highlights the involvement of non-motor areas in ALS. Kuruvilla and colleagues assessed individuals with ALS and HC on two N-back working memory tasks [87]. Decreased activation located approximately over the medial prefrontal cortex in both hemispheres was reported in ALS, while cognitive performance was relatively intact. This finding led the authors to conclude that compensatory reorganization and resource reallocation from other cortical regions may occur in ALS in order to meet cognitive demands when prefrontal neurons degenerate. Moreover, unlike in the HC, activation did not increase with escalating task difficulty. Therefore, it was speculated that increasing task difficulty exceeds the compensatory capabilities in ALS patients.

## 4. Discussion

This review highlighted that cortical activation as measured with fNIRS is influenced by the underlying neuropathological entity, the degree to which neural networks have been compromised (early, advanced), and the complexity and target of clinical assessments (neuropsychological, motor, and dual tasks). Even among individuals afflicted with the same neurodegenerative disorder, there is a significant heterogeneity in cortical activation patterns. These patterns may reflect either adaptive or maladaptive pathophysiological processes. 

Compensatory responses usually occur early in the course of neurodegeneration [10]. During the initial stages, patients often maintain a normal or nearly normal level of clinical performance discrepant to the degree of structural compromise [88]. In the course of neurodegeneration, the existence of a crucial breakpoint is theorized, whereby the early pattern of normal (or nearly normal) clinical performance is succeeded by the more typical pattern of clinical impairment [11]. The earlier stage is dependent on the development and recruitment of neurovascular compensatory mechanisms, whereas the later stage represents the failure of compensatory responses. This sequence is often captured by functional neuroimaging as increased cerebral perfusion (higher metabolic needs required for the preservation of normal functions) followed by decreased brain perfusion (limited metabolic needs due to more severe neurodegeneration) [11]. Hemodynamic responses (and, in turn, patterns of cortical activation and neural compensation or degeneration) can be quantified with fNIRS [12,13]. 

Based on the above, the relative maintenance of cognitive and/or motor functions along with greater hemodynamic responses (hyperactivation) in fNIRS suggest the involvement of compensatory mechanisms [89]. Compensatory processes appear to be inversely related to the degree of neurodegeneration (as is apparent in early disease stages), whereas failure of neural compensation (reduced hemodynamic responses—hypoactivation) is predominantly observed at more severe stages [37]. The latter is particularly evident when shifting from simple to complex and/or dual tasks, exposing the inability of patients to cope with excess cognitive load and the utter disruption of compensatory responses. 

Our findings indicate that the prefrontal cortex of individuals with MCI (with a rightward shift of prefrontal recruitment) may engage neural compensatory mechanisms to support declining brain functions early in the course of the disorder. However, as neurodegeneration progresses, compensatory mechanisms are compromised; therefore, although both cortical hyperactivation and hypoactivation patterns have been revealed in MCI, early AD is consistently characterized by hypoactivation patterns. In other words, MCI patients may be able to handle increasing cognitive load using compensatory mechanisms at first until they reach their cognitive capacity limits for neural compensation due to more severe neurodegeneration or more demanding cognitive tasks. Individuals with different MCI subtypes and levels of cognitive impairment subjected to heterogeneous cognitive assessments (targeting different cognitive domains and bringing in different cognitive workloads) are expected to reveal distinct patterns of cortical activation. Similarly, discrete underlying pathologies are anticipated to introduce heterogeneous activation patterns (with potential implications for their differential diagnosis—see the FTD paradigm). Of note, the analysis of fNIRS data in PD provided evidence of the leveraging of prefrontal cognitive resources (especially executive function) for the compensation of locomotor impairments as well.

The main limitation of this review is the small number of published studies featuring small and often heterogeneous samples of participants. Second, the majority of the studies primarily focused on task-related hemodynamic responses in frontal and prefrontal areas. This tendency of authors to focus primarily on frontal operations and executive function limits the potential value of fNIRS in clinical practice. It is possible that, due to brain plasticity, the mental workload is shifted to other areas of the brain during task performance. One likely area that was not monitored by most studies is the parietal cortex. Future studies ought to include larger and more homogeneous samples, provide more comprehensive evaluations, and report their findings with accuracy and transparency [90,91]. fNIRS itself exhibits a number of limitations related to spatial resolution (restricted to the outer cortex), interference of extracranial matter with measurements (muscles, skull, dura, etc.), the lack of standardized processes (implementation and analysis), and the inability to extract absolute hemoglobin concentrations [21]. Also, abrupt head movements and misplacement of the diodes may lead to measurement errors (artifacts are not corrected automatically by software) [92]. Upcoming technological advancements are expected to optimize this promising technique and establish fNIRS as a useful tool in the fields of research and clinical practice. 

## 5. Conclusions

Considering the impact of neurodegenerative disorders, it is necessary to develop widely accessible and easily operated tools to overcome early diagnostic challenges [93,94,95]. Thanks to its user-friendly nature and compatibility with other functional neuroimaging techniques, fNIRS holds great potential for the diagnostic assessment of early neurocognitive and motor decline. Early detection serves research purposes and facilitates timely intervention, better management, and the minimization of iatrogenic complications [49,96,97]. Moreover, the detection of preserved neuroplasticity offers opportunities for personalized rehabilitation, which can address individuals’ needs more efficiently. However, further research is needed to integrate fNIRS and determine its exact place in clinical practice.

## Figures and Tables

**Table 4:** fNIRS studies involving older adults with ALS and HC.

Study	Participants	Clinical Scores	Mean Age in Years ± SD	Tasks [Corresponding Functions]	fNIRS Data [Mentioned Involved Areas]	Conclusions
Deligani et al., 2020 [83]	Definite ALS (*n* = 10) HC matched for age (*n* = 9)	ALSFRS-R: 23.2 ± 13.7	58.2 ± 11.6 (ALS) 61.0 ± 3.8 (HC)	None/Resting state with closed eyes	Frontal and right prefrontal regions: Increased functional connectivity in the ALS group.	The increased resting-state functional connectivity in ALS patients is likely a compensatory mechanism for monitoring deficits.
Borgheai et al., 2019 [84]	ALS (*n* = 6) HC matched for age (*n* = 12)	ALSFRS-R: 11.6 ± 9.5 Average ALS-CBS: 90.5 ± 6.9	57.0 ± 15.7 (ALS) 56.4 ± 15.4 (HC)	Oddball-based dual visual–mental task [Arithmetic operations: Arousal, numerical representation, mental arithmetic, logical thinking Visuospatial oddball paradigm: visuospatial and decision-making ability]	Left prefrontal area corresponding to the dorsolateral prefrontal cortex: Greater reduction in HbO in the ALS group—different temporal patterns of HbO responses in ALS and the HC.	These findings suggest the involvement of non-motor areas in ALS (prefrontal cortex).
Kopitzki et al., 2016 [86]	Definite or probable ALS (*n* = 31) HC matched for age and gender (*n* = 30)	ALSFRS-R: 36.5 ± 5.4 DPR: 0.5 ± 0.37	61.4 ± 12.1 (ALS) 62.6 ± 9.9 (HC)	None/Resting state with closed eyes	No significant difference in homotopic resting-state functional connectivity (rs-FC) between ALS patients and the HC. ALS patients displayed an altered correlation between homotopic rs-FC values obtained at different cortical sites when compared to the HC. Homotopic rs-FC of the anterior temporal lobes correlated with ALS-specific white matter degeneration of the corpus callosum and corticospinal tract, as well as with the rate of motor decline in ALS patients without executive dysfunction.	ALS patients displayed an altered spatial pattern of correlation between homotopic rs-FC values measured in different non-motor-associated cortical areas when compared to the HC. These findings suggest the involvement of non-motor areas in ALS. fNIRS-derived measures may be a sensitive neural marker for detecting early neurodegenerative changes in non-motor areas.
Ayaz et al., 2014 [85]	ALS (*n* = 17) HC (*n* = 17)	ALSFRS-R: 35.06 ± 6.56 PBAC: 78.41 ± 5.95 (ALS), 80.35 ± 4.51 (HC)	57.3 ± 7.5 (ALS) 55.1 ± 6.3 (HC)	Number Interference Task (NIT) [Executive functions (Inhibitory control, Cognitive flexibility)] King–Devick Task (KDT) [Attention, Inhibitory control, Processing speed, Language processing, Oculomotor function] Continuous Performance Task (CPT) [Sustained attention and inhibitory control]	Lateral and medial prefrontal cortex regions, inferior frontal gyrus: Higher activation in ALS during tasks. Highest activation at the beginning of NIT for the ALS group and progressive reduction of activation with subsequent trials of increasing difficulty—contrary to the HC.	A higher neural cost of task initiation was found in the ALS group. Increasing the task’s difficulty exceeds the compensatory capabilities in patients with ALS.
Kuruvilla et al., 2013 [87]	Non-demented ALS (*n* = 5) HC matched for age and gender (*n* = 5)	ALSFRS-R: 36.8 ± 7.98 MoCA: 22.6 ± 1.82 (ALS), 26.3 ± 0.96 (HC)	60.2 ± 15.09 (ALS) 59.2 ± 14.13 (HC)	N-Back task (1-back to 3-back task) [Working Memory]	Location approximately over the medial prefrontal cortex in both hemispheres: Reduced activation in the ALS group during N-back tasks. Increasing activation in the HC group with increasing task difficulty. Decreased activation in the ALS group not affected by increasing the task’s difficulty.	Reduced prefrontal activity despite intact behavioral performance for a working memory task may suggest compensatory reorganization and resource reallocation from other cortical regions in order to meet cognitive demands when prefrontal neurons degenerate. Increasing the task’s difficulty exceeds the compensatory capabilities in patients with ALS.

Note: Quantitative data are presented as the mean ± standard deviation depending on the data presentation in the respective article; fNIRS: functional near-infrared spectroscopy; ALS: amyotrophic lateral sclerosis; HC: healthy controls; ALSFRS-R: ALS Functional Rating Scale—Revised; CBS: Cognitive Behavioral Screen; DPR: disease progression rate; PBAC: Philadelphia Brief Assessment of Cognition; MoCA: Montreal Cognitive Assessment.

## Data Availability

Not applicable.
